# A Molecular Study of Microbe Transfer between Distant Environments

**DOI:** 10.1371/journal.pone.0002607

**Published:** 2008-07-09

**Authors:** Sean D. Hooper, Jeroen Raes, Konrad U. Foerstner, Eoghan D. Harrington, Daniel Dalevi, Peer Bork

**Affiliations:** 1 Department of Energy Joint Genome Institute (DOE-JGI), Walnut Creek, California, United States of America; 2 EMBL, Heidelberg, Germany; 3 Biological Data Management and Technology Center, Lawrence Berkeley National Laboratory, Berkeley, California, United States of America; NERC Centre for Ecology and Hydrology, United Kingdom

## Abstract

**Background:**

Environments and their organic content are generally not static and isolated, but in a constant state of exchange and interaction with each other. Through physical or biological processes, organisms, especially microbes, may be transferred between environments whose characteristics may be quite different. The transferred microbes may not survive in their new environment, but their DNA will be deposited. In this study, we compare two environmental sequencing projects to find molecular evidence of transfer of microbes over vast geographical distances.

**Methodology:**

By studying synonymous nucleotide composition, oligomer frequency and orthology between predicted genes in metagenomics data from two environments, terrestrial and aquatic, and by correlating with phylogenetic mappings, we find that both environments are likely to contain trace amounts of microbes which have been far removed from their original habitat. We also suggest a bias in direction from soil to sea, which is consistent with the cycles of planetary wind and water.

**Conclusions:**

Our findings support the Baas-Becking hypothesis formulated in 1934, which states that due to dispersion and population sizes, microbes are likely to be found in widely disparate environments. Furthermore, the availability of genetic material from distant environments is a possible font of novel gene functions for lateral gene transfer.

## Introduction

The advances of environmental sequencing projects, or *metagenomes*, have brought methods and concepts from molecular biology and comparative genomics to the field of microbial ecology. Many of the same tools that are used in the analysis of isolate genomes can now be applied to whole communities of organisms [1]. In this work, we perform what can be described as comparative metagenomics, where we attempt to identify genetic material that originated from outside the environment, possibly transported by physical processes such as wind or water. For instance, dust clouds may carry microbes over vast distances [2], and carrier organisms such as birds and humans [3] are potential vehicles for transporting microbes. In other cases, drainage from cultured soils may pollute water [4], and it is conceivable that microbes may be transferred in the process.

The motility and sheer numbers of microbes form the basis for the Baas-Becking hypothesis formulated in 1934 [5]. It can be summed up as follows; everything is everywhere and the environment selects. For instance, the hypothesis implies that there is a good chance of finding trace amounts of a wide range of bacterial species wherever we look, but this does not mean that the species will grow or even survive in its new environment. Even if the transported microbe is inert, it would still contribute its genome (and DNA) to the new environment. Thus, the transported DNA may remain packaged within an inert host, within a surviving host, or may be free as the result of a ruptured or digested cell. Free-form DNA has been observed in for instance ocean sediments [6], where it comprises up to 90% of all DNA.

Regardless of the fate of the specific microbe, its DNA can be captured and detected at the time of a metagenomic sampling. Depending on the frequency of the DNA, reads will assemble into contigs or appear as single-reads, and can then be analysed computationally. In this work, we examine two such metagenomes: the Minnesota farm soil [7] data set and the Sargasso sea [8] data set, and attempt to evaluate the interchange, if any, of microbes between them using DNA sequences as proxies. Thus, we will evaluate the Baas-Becking hypothesis by examining the proportion of sequences that *i)* appear very different from other reads in its set and *ii)* appear more similar to reads in the other set. We will then study those sequences to which are potentially results of a microbe transfer across environments.

This comparative process is conceptually very similar to the study of lateral transfer genes (LGT) in isolate genomes. There is an extensive literature describing this approach, from early but seminal studies using atypical nucleotide composition as indicators of LGT [9]–[11] to extensive phylogenetic studies covering hundreds of genomes [12]. All approaches require a careful choice of characteristics to use as discriminators of whether a sequence appears to be typical or not for its genome. We will substitute genomes for metagenomes in this study, so special attention must be given to the choice of discriminators.

We chose three distinct characteristics as discriminators; two nucleotide composition measures and one protein orthology measure. The first measure is based on the guanine/cytosine (GC) content of the sequence. GC content has been found to vary not only between species but also between environments [13]. For the farm soil and Sargasso sea data sets (hereafter referred to as *soil* and *sea*), we observe clear differences in the overall GC content. Soil has a high GC content at 61%, compared to only 34% in sea [13]. This difference is even more pronounced when comparing only the synonymous third codon position of genes (hereafter GC3s%) which avoids selection on the protein level. The more pronounced differences in GC3s% than GC suggest a mutational pressure on the choice of base exerted by exogenic factors, as previously described [13].

The second measure is based on oligomer frequency patterns (OFPs; [14]–[16]). For instance, the OFP of the oligomer TTATA, relative to the occurrences of T and A respectively, differs widely between organisms.One of the first systematic studies reported showed that the composition of dimers is conserved within genomes but different between genomes [17]. Since then many different methods have been developed to capture the genomic signature of bacteria and they have been use widely for either binning of metagenomic data [18] or the identification of lateral gene transfer [19].

Superficially, it could be assumed that GC3s% could be included in this measure, but the level of information is distinctly different in three aspects. Whereas GC3s% directly measures the mutational pressure, the OFP measures the effect of mutational context biases. Since OFPs are also normalized by nucleotide content, this measure is largely independent of GC3s%. Finally, since we study more than one base, OFPs are a more sensitive discriminator.

The third measure is based on protein similarity between translated open reading frames in both data sets. The rationale is that if a gene in e.g. soil has a substantially higher level of orthology to proteins in sea, compared to the rest of the proteins in soil, then it is less likely to be a common fixture of soil. If the two environments never interchange material, then we would expect high levels of orthology only for genes coding for highly conserved and ubiquitous functions, such as cell machinery. However, if a transfer of microbes occasionally occurs between soil and sea, we would expect to find non-ubiquitous yet highly orthologous genes.

For each of these discriminators individually, criticisms can be raised. For instance, bacteria which are parasites within soil eukaryotic cells may essentially live in a mini-environment similar to that of sea microbes, possibly resulting in similarities in GC3s%. Furthermore, organisms that are only distantly related but have similar DNA repair mechanisms could appear similar in OFPs. Orthology may also be spurious due to strict conservation of amino acid sequences of proteins, or by random chance.

Despite individual concerns such as those listed above, it becomes increasingly difficult to regard these open reading frames as false positives when all three discriminators are fulfilled.

In this work, we apply the three discriminators to predicted genes in the soil and sea sets in order to find genes that are consistent with an interchange of microbes between environments. This transfer of microbes did not specifically occur from the Minnesota farm soil to the Sargasso sea or vice versa, but from environments which share features with either the farm soil or Sargasso sea data. As both GC content and protein composition correlate with the similarity of environments [7], [13], it is reasonable to assume that our three discriminators also account for transfers from environments that are at least geographically close to the sampling points or are of similar consistency [20], [21].

## Results

**Figure 1:**
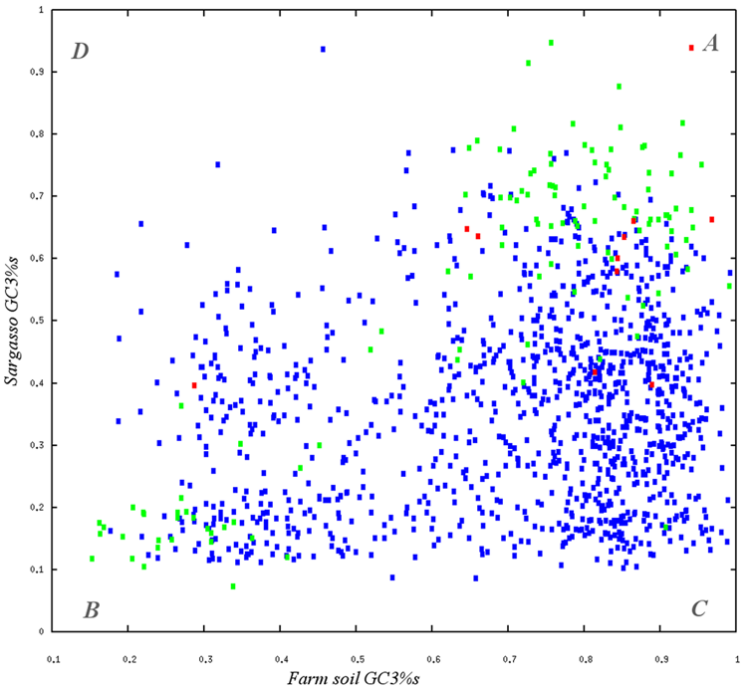
GC3s distribution of orthologous Genes. Distributions of GC3s for each of 1216 ORF pairs with closer similarity in the foreign environment. Using GC3s% = 48% as a separator (dotted lines), the ORF pairs are classified based on the GC content of its two members. Category A (upper right) is the quadrant where we expect to find possible transfer events from soil to sea, since these pairs have high GC3%s values for both members. Pairs in category B (lower left) have low GC3%s scores for both Genes, which could suggest a transfer from a sea-like environment to soil. Category C (lower right) has typical GC3s% values for both members of ORF pairs. These pairs are likely to be ancient conserved sequences. Finally, Category D (upper left) has atypical values for both Genes, close to the expected given the shape of the GC3% distribution (28 observed, 24 expected). Unsaturated Ks values are green, and pairs with Kn/Ks>1 are red.

Starting with 184,000 genes in the soil set [7] and 700,000 genes in samples 2–4 from the sea set [8], we identified 1,216 genes that have a closer hit in the foreign environment than their own. These genes, together with their match in the foreign environment, formed pairs which allowed us to compare their features. To classify whether the GC content of these candidate gene pairs is endogenous in one but atypical in the other environment, we used the average of the two environmental GC3s% averages (48%) as a breakpoint (Fig. 1).

Of the 1216 ORF pairs, 284 sea genes had atypical GC3s% values (>48%); a strong over-representation both in absolute terms and in significance (*p<10^−13^*), when compared to the expected number of 109 (based on the proportion of all sea genes with atypical GC3%s values). Conversely, the over-representation of soil genes with GC3s%<48% is not as strong, yet significant: 221 compared to an expected 174 (*p<10^−3^*).

Quadrant A (Fig. 1) thus represents gene pairs where the soil genes have typical GC3s% values and the sea genes have GC3s% values much higher than the sea average. Accordingly, quadrant B has lower than average soil GC3s% and typical sea GC3s%. Overall, quadrant A has 170% as many pairs as expected and B 127%. However, since sample sizes are unequal, we subsampled the sea set 10 times into random subsamples of a size roughly equal to soil (Table 1). The degrees of over-representation remained at 165% and 123% respectively. Details of quadrants A–D are provided as supplementary Tables S1–S4.

**Table 1:** Resampling of sea set.

S	nA (74)	nB (117)	aA	aB	sA	sB	sA/sB
1	123	139	5	0	34	17	2.00
2	123	133	2	1	26	15	1.73
3	120	143	4	1	32	16	2.00
4	137	140	3	1	33	20	1.65
5	126	147	4	0	27	19	1.42
6	107	151	2	1	23	19	1.21
7	111	147	4	2	35	23	1.52
8	114	140	3	0	39	21	1.86
9	131	142	7	0	29	13	2.23
10	130	162	4	0	38	21	1.81
Full set	284	221	8	1	87	31	2.81

Distributions of genes in quadrants A and B. Key: **S**: sample number, **nA**; number of gene pairs in A, with the average expected number in parenthesis, **nB**; number of gene pairs in B, also with expected in parenthesis, **aA**; number of gene pairs in A with *Kn/Ks*>1, **aB**; number of gene pairs in B with *Kn/Ks*>1, **sA**; number of gene pairs in A with *Ks*<2, **sB**; number of gene pairs in B with *Ks*<2.

**Figure 2:**
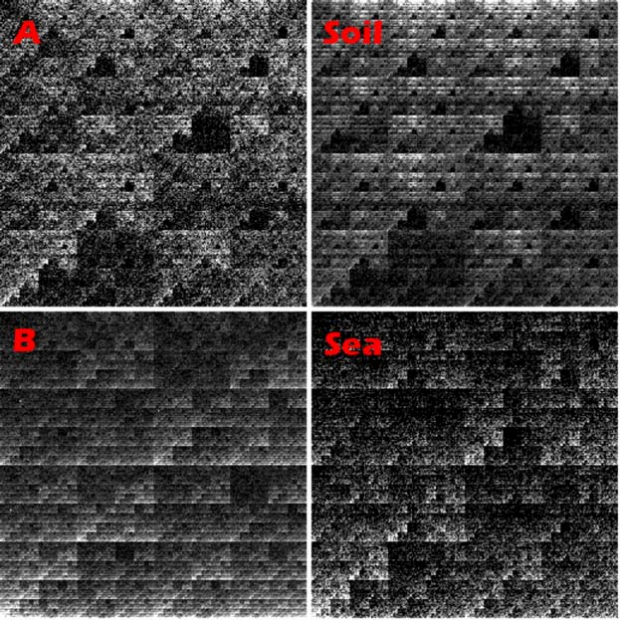
Chaos Game Representation (CGR) plot of oligomer frequencies of A and B vs soil and sea patterns. Note the similarities between A and soil, and B and sea respectively. Figure intensities have been normalized for clarity. CGR plots are a way of visualizing chain processes, such as oligomer patterns. See methods for details.

At this point, we have created three classes of genes using orthology and GC3s% as discriminators. These classes represent genes that may have been transferred into a new environment (quadrants A and B) or simply conserved genes (quadrant C). If all three classes are actually false positives, we would expect OFPs to be distributed according to random expectation, i.e. soil genes would have OFPs similar to the soil set in large, and analogously for sea genes. Out of 16,450 random soil genes, 14,040 map to soil (85.3%). For sea, 14,257 of 16,476 map correctly to sea (86.5%). Thus, we expect that of the 284 soil genes in A, 242 should map to soil. However, we observe that only 53 soil genes map better to soil than sea. This is a strong under-representation (*p<10^−137^*). In quadrant B, we expect that 191 of the 221 sea genes would map to sea, but observe only 13 (*p<10^−161^*). The OFPs of quadrants A and B, compared to soil and sea sets are visualized as chaos game representations ([22]; see methods) in Fig. 2.

Finally, as an additional control, we study genes in quadrant C, which we do not believe to be transferred. Here, we find 674 of 678 unique sea genes and 579 of 663 unique soil genes mapped to their own environments. The sea mappings are actually significantly over-represented (*p<10^−14^*), and soil genes map to soil about as often as expected.

The results strongly imply that genes in quadrants A and B are not only atypical in their current environments, but also highly similar to the external and internal mutational pressures in the other environment.

Amino acid identity is a measure of the similarity between the amino acid translations of genes, and as such focuses on the non-synonymous bases. A further comparison would be to measure the synonymous substitution rate *Ks* [23] of a gene pair, since this rate quickly becomes saturated over time. A *Ks* value of over 2.0 suggests that each base has on average been substituted at least once; changes are therefore saturated. However, values of less than 2.0 suggest a higher level of similarity than evident from amino acid identities. Finding such unsaturated pairs in the quadrants would further suggest a shared recent history. In quadrant A we find 87 such pairs, and in B 31. Again, this is not what we expect if we consider the gene pairs to simply be ancient homologs. In quadrant C for instance, which we consider to be composed mainly of ancient homologs, we find only 8 unsaturated pairs out of 667. None score lower than 1.2. Thus, unsaturated gene pairs are significantly overrepresented in quadrants A and B (both at *p<10^−14^*). Furthermore, the ratios (87 to 31) again suggest a bias in directionality from soil to sea. This also holds when the datasets are resampled (Table 1), suggesting that it is not an effect of sample sizes.

If genes in quadrants A and B are the results of microbes, alive or not, traversing large distances between soil and sea, then it would be interesting to know which species they come from. Determining which taxa are included in a metagenome is referred to as *binning*, and is not a straight-forward task. Since the focus of this paper is not on binning Minnesota farm soil and Sargasso, we employ a simple best hit approach and record the species for each gene. The results were then mapped onto the Interactive Tree of Life [24] and are available as supplementary figures (Fig. S1–S3).

For quadrant A, which should represent transfer from soil to sea, we find a relatively even contribution from a wide range of phyla and a considerable (∼25%) contribution from the Rhizobium/Bradyrhizobium clades. Both of these families are predominantly terrestial bacteria, which is consistent with our findings.

Similarly, in quadrant B, we also observe an even contribution from a wide range, with a stronger representation from the bacteroides genus. Bacteroides are not predominantly soil bacteria, but can be found in the guts of farm animals. It is hence not subject to the mutational pressures of soil but is readily and consistently transferred from animals to soil. It is therefore not inconceivable that the contribution of bacteroides may be via animal waste to soil, and then to sea. This does not weaken our results, but rather strengthens the conclusion that transfer is predominantly from soil to sea rather than vice versa, and underlines the interaction of diverse environments other than the two we have studied.

For comparison, we include best hit binnings for the whole soil and sea sets (Fig. S4–S5). Noteworthy is the huge dominance of *Candidatus pelagibacter* (e.g. [25]) in the full sea set, but which is largely absent in quadrant A. Furthermore, our simplistic binning approach suggests that there is no strong contribution from any potential lab contaminant.

### Protein function

The null hypothesis is that the transferred DNA is selected randomly, and therefore codes for random products. Supplementary Table S5 illustrates the distribution of protein functions by Cluster of Orthologous Gene categories [26]. Generally, quadrants A, B and C are consistent with a random selection of functions drawn from the distributions of the whole metagenome sets, but some differences nonetheless stand out. Quadrant B has lower numbers of ORFs coding for energy production and conversion and general function prediction (COG category C and R) than quadrants A and C, but higher numbers of ORFs coding for translation, replication and repair.

### Fate of transfers

Based on our studies, quadrants A and B are consistent with a transfer effect. But what of the fate of these transported genes or DNA fragments? Most likely they will simply be degraded, but there is also a possibility of incorporation into indigenous genomes, constituting a true LGT. We can first study the GC content of the flanking DNA or neighboring gene, if any, and see if it is different or similar. Of the 505 candidates, the majority of flanking DNA has similar GC values. This suggests that large regions (likely entire genomes, plasmids or chromosomes) have been transferred but not assimilated. In quadrant A however, 31 of 284 sea genes have one or more neighbors with a GC3s%<48%, which may suggest that some genes are occasionally integrated into indigenous genomes. In this case, the ability to assemble contigs with several genes also suggests that these genes may have been incorporated into abundant species. Furthermore, we studied transfer candidates that seem to be under positive selection, as this would indicate an adaptation to the new environment and therefore LGT. We find 8 ORF pairs that suggest an accelerated evolution (*Kn/Ks*>1): 7 in quadrant A and 1 in B. The annotated functions [26] of these ORFs in the process of adaptation are diverse (Tables S1–S2). However, the function that is under selection is not necessarily the same as the annotated function [27] so we cannot exclude a common functional theme due to the process of adaptive radiation [28]. Unfortunately, only 2 of the 7 ORFs have neighbors – both with similar GCs% values. This would suggest that these adapting ORFs may have been incorporated along with other genes which are not under selection in the new environment. Moreover, given the high rate of amelioration at the synonymous base, it is likely that many such ORFs would have *Kn/Ks*<1 despite adaptation. These 8 ORFs are therefore a conservative estimate.

## Discussion

### Microbe transfer

Through several different comparisons, we have found a set of genes, however small, for which the simplest explanation is microbe transfer. Specifically, we seem to detect a transfer of genetic material in the Sargasso samples from an environment very similar to Minnesota soil and vice versa. Given the prodigious population sizes and motility of e.g. bacteria, it should not be a surprising conclusion. However, detecting it is not as intuitive, and we believe we are the first to address this question using computational methods.

Our data is for natural reasons limited; other oceanic and soil samples may contain other transfer candidates, and the total transfer to the Atlantic Ocean from environments similar to Minnesota farm soil must be considerably larger. However, we believe it is an informative snapshot given the current data. With more large-scale sequencing projects, the picture will undoubtedly improve.

We also suggest a bias in transfer from soil to sea, in line with the generally accepted flow of water from land to oceans. Furthermore, the presence of bacteriodiales in quadrant B further tilts the scale in favor of transfer from soil to sea, since they are likely to have originated in a third environment – animal gut. Assessing the total proportion of foreign DNA in a metagenome is a difficult task at best. In this study, we focused on sets with quite different nucleotide compositions, which simplify detection of foreign DNA. Other sources of transfer may be more similar to the receiving environment, and detection is therefore more complicated. In addition, rare foreign DNA may be present in low numbers and is likely to evade detection by normal shotgun sequencing. Thus, in the case of transfers between soil and sea, the amount of transferred DNA seems to be abundant enough to be detectable by shotgun sequencing, even though it is only a fraction of the amount of indigenous DNA.

This study therefore suggests that the species abundance distributions of metagenomes which are not physically isolated may have exceedingly long ‘tails’ composed of rare organisms. It is therefore unlikely that sequencing projects of this type will reach full coverage in the near future.

### Consequences for LGT

While little data is available on genes which have been incorporated into new hosts, our findings suggest that it is possible. Furthermore, it has been found that the extent of LGT in metagenome samples is comparable to that of isolate genomes [29], suggesting that LGT is an active process also within the soil and sea microbiomes. Combined with our findings, we suggest that the impact of LGT could be more far-reaching than previously thought, since functions need not be acquired from the immediate vicinity but from entirely different environments. This would also include non-microbial donors, such as genetically modified plants.

## Materials and Methods

Our approach employs three basic discriminators to assess microbe transfer and is based on the study of lateral gene transfer. First, we test genes for their orthology against the other environment. If a gene in either set has a higher (20% better) homology score to an gene in the foreign environment than to its own, we select that gene pair for further investigation. Furthermore, all orthology must fulfil at least 80% protein similarity over at least 90% of the shortest gene. Genes under 100 base pairs in length were ignored. As a second measure, we calculated the GC content at the synonymous base (GC3s%). Using the GC3s% values of each member of a pair, we then classified pairs into three major categories depending on if one or no member had GC3s% values atypical for their environment. GC3s% was calculated using *codonw* (http://codonw.sourceforge.net/). As a third measure, oligomer frequencies were calculated using *softPSTk-Classifier* [15]. To further stress that the genomic signature of oligomers is not simply a result of the difference in GC between the two environments, we decided to also show the visualization using chaos game representations (CGR) plots [22]. The points in these graphs can easily be calculated recursively using the relation,
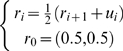
where
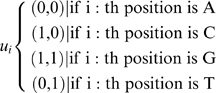



DNA with different composition will end up with different coordinates in the plot depending on the symbols. All points are bound to the unit square. Plot intensities have been normalized for clarity. Note that no conclusions have been drawn directly on the figure itself, rather from significance tests of the distributions in the quadrants. The figure is included for visualization purposes only.

### Environmental data

We used the same data from Sargasso and Minnesota as were used previously by Tringe and co-workers [7]. Note that this data set does not include sample 1 from Sargasso, due to recent criticism [30].

Gene predictions were performed by the original authors, resulting in roughly 700 000 and 184 000 ORFs respectively.

### Estimation of synonymous (Ks) and nonsynonymous substitution rates (Kn)

Nucleotide sequences were pairwise aligned by ClustalW [31] using the corresponding protein sequences as an alignment guide. Gaps and adjacent divergent positions in the alignments were removed. *K_S_* estimates were obtained with the Codeml [23] algorithm in the PAML package (F3x4 model, gamma shape parameter and transition-transversion ratio estimated from the data [32]). Calculations were repeated five times to avoid incorrect *Ks* estimations due to suboptimal local maxima.

## Supporting Information

Table S1 ORF pairs belonging to category A. Sea: Sargasso ORF. Soil: Minnesota ORF. ID: protein identity. Pos: protein positive similarity. Length: Overlap length of overlapping sequence. GC_sea: GC3s% of Sargasso ORF. GC_soil: GC3s% of Minnesota ORF. Diff: GC3s% difference. KaKs: substitution ratio of synonymous to non-synonymous base. Ka: synonymous base substitution rate. Ks: non-synonymous substitution rate. COG: COG assignment. Func: COG functional category. Annotation: predicted function.
(0.04 MB CSV)

Table S2 ORF pairs belonging to category B. Sea: Sargasso ORF. Soil: Minnesota ORF. ID: protein identity. Pos: protein positive similarity. Length: Overlap length of overlapping sequence. GC_sea: GC3s% of Sargasso ORF. GC_soil: GC3s% of Minnesota ORF. Diff: GC3s% difference. KaKs: substitution ratio of synonymous to non-synonymous base. Ka: synonymous base substitution rate. Ks: non-synonymous substitution rate. COG: COG assignment. Func: COG functional category. Annotation: predicted function.
(0.03 MB CSV)

Table S3 ORF pairs belonging to category C. Sea: Sargasso ORF. Soil: Minnesota ORF. ID: protein identity. Pos: protein positive similarity. Length: Overlap length of overlapping sequence. GC_sea: GC3s% of Sargasso ORF. GC_soil: GC3s% of Minnesota ORF. Diff: GC3s% difference. KaKs: substitution ratio of synonymous to non-synonymous base. Ka: synonymous base substitution rate. Ks: non-synonymous substitution rate. COG: COG assignment. Func: COG functional category. Annotation: predicted function.
(0.10 MB CSV)

Table S4 ORF pairs belonging to category D. Sea: Sargasso ORF. Soil: Minnesota ORF. ID: protein identity. Pos: protein positive similarity. Length: Overlap length of overlapping sequence. GC_sea: GC3s% of Sargasso ORF. GC_soil: GC3s% of Minnesota ORF. Diff: GC3s% difference. KaKs: substitution ratio of synonymous to non-synonymous base. Ka: synonymous base substitution rate. Ks: non-synonymous substitution rate. COG: COG assignment. Func: COG functional category. Annotation: predicted function.
(0.00 MB CSV)

Table S5 A breakdown of COG categories by quadrant. The ‘expected’ occurence is based on the classification of the full soil and sea sets Cat: COG category. A,B,C: quadrants. Expected: expected number given random occurrence.
(0.00 MB CSV)

Figure S1 Phylogenic distribution of category A.
(18.08 MB TIF)

Figure S2 Phylogenic distribution of category B.
(18.08 MB TIF)

Figure S3 Phylogenic distribution of category C.
(18.08 MB TIF)

Figure S4 Full phylogenic distribution of the Sargasso set.
(18.08 MB TIF)

Figure S5 Full phylogenic distribution of the soil set.
(18.08 MB TIF)
