# Supplementary figures and images for: A Molecular Study of Microbe Transfer between Distant Environments
Source: PLoS One. 2008 Jul 9;3(7):e2607. doi: 10.1371/journal.pone.0002607 (PMC2442867; doi:10.1371/journal.pone.0002607)

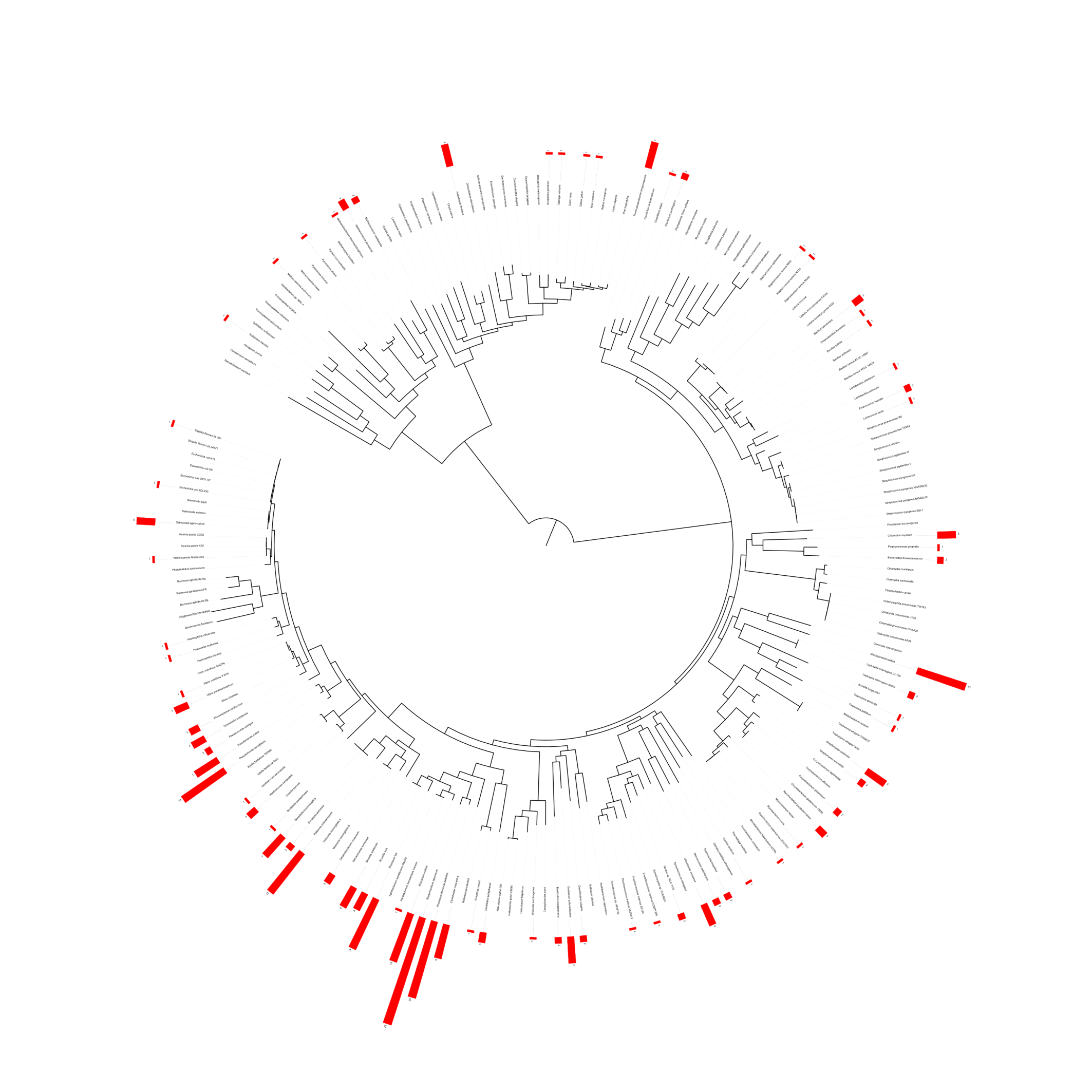

Supplement: Figure S1 — Phylogenic distribution of category A. (18.08 MB TIF) [file pone.0002607.s006.tif]

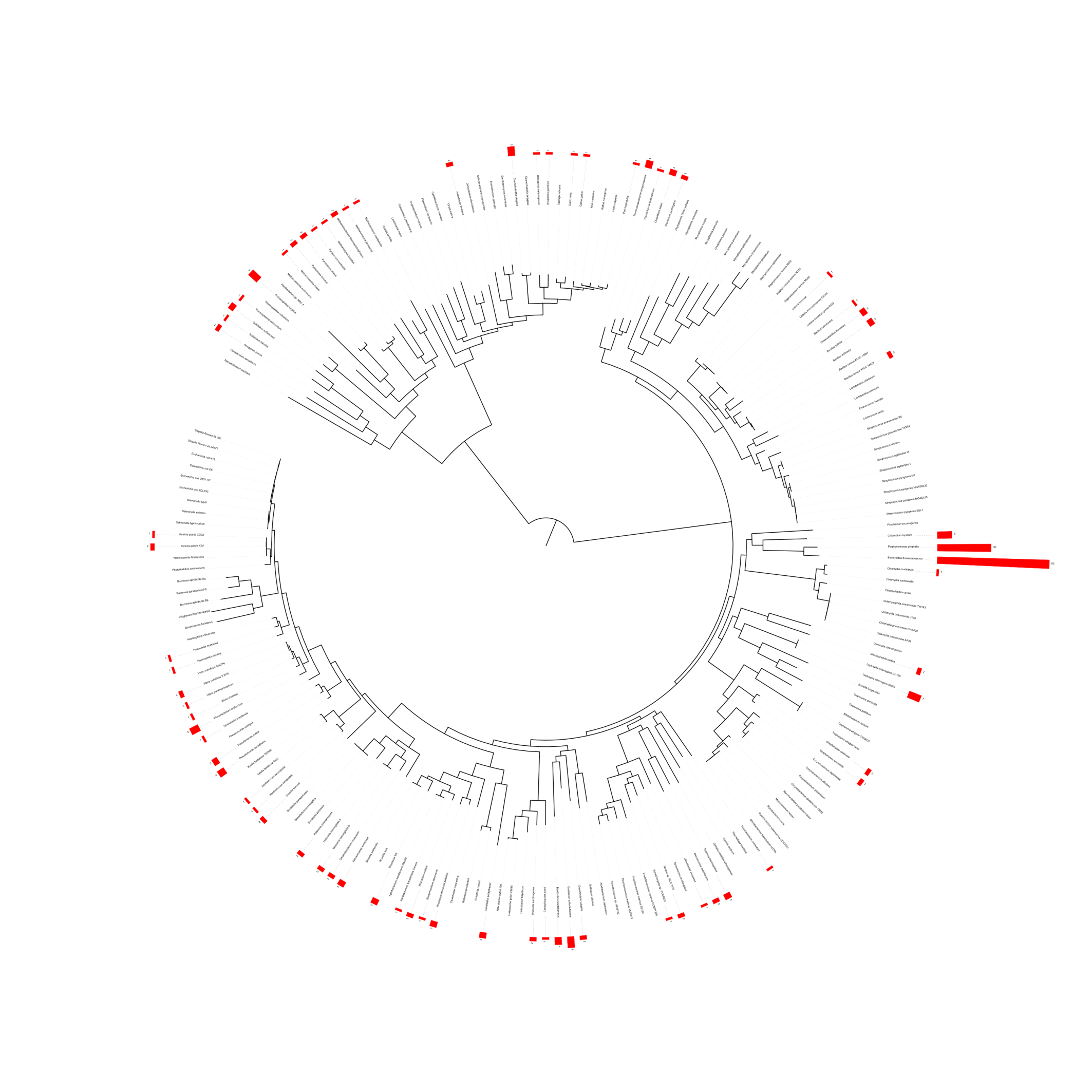

Supplement: Figure S2 — Phylogenic distribution of category B. (18.08 MB TIF) [file pone.0002607.s007.tif]

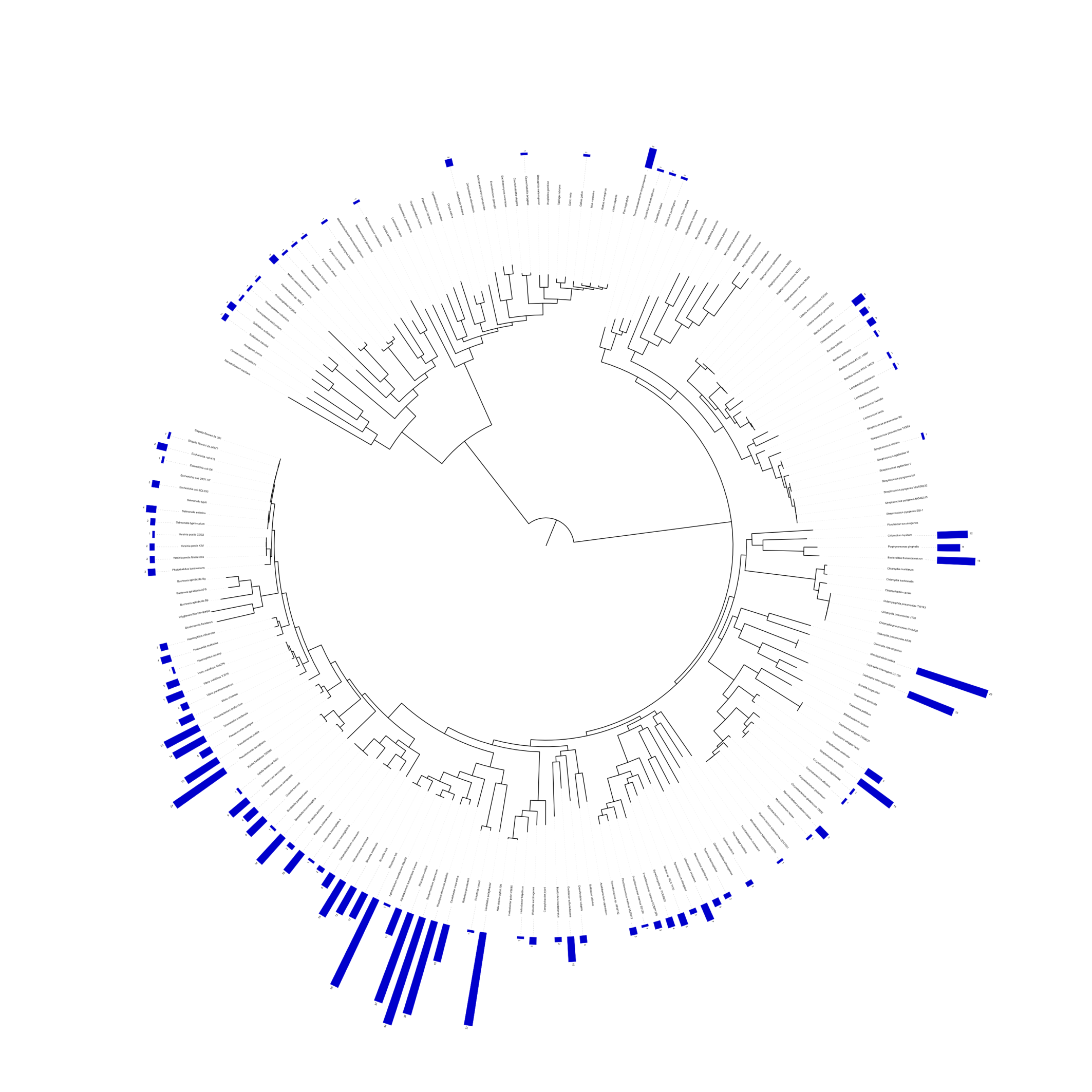

Supplement: Figure S3 — Phylogenic distribution of category C. (18.08 MB TIF) [file pone.0002607.s008.tif]

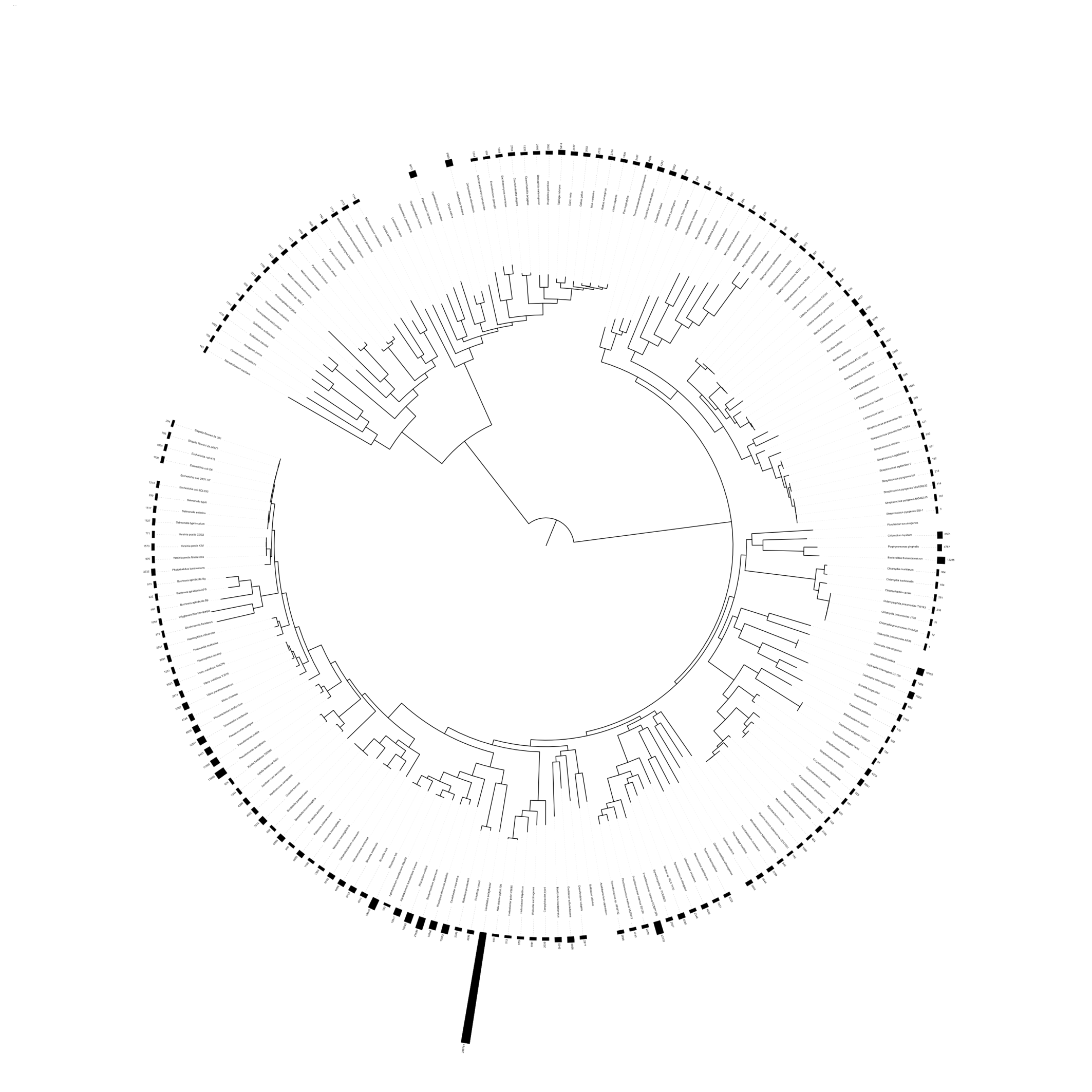

Supplement: Figure S4 — Full phylogenic distribution of the Sargasso set. (18.08 MB TIF) [file pone.0002607.s009.tif]

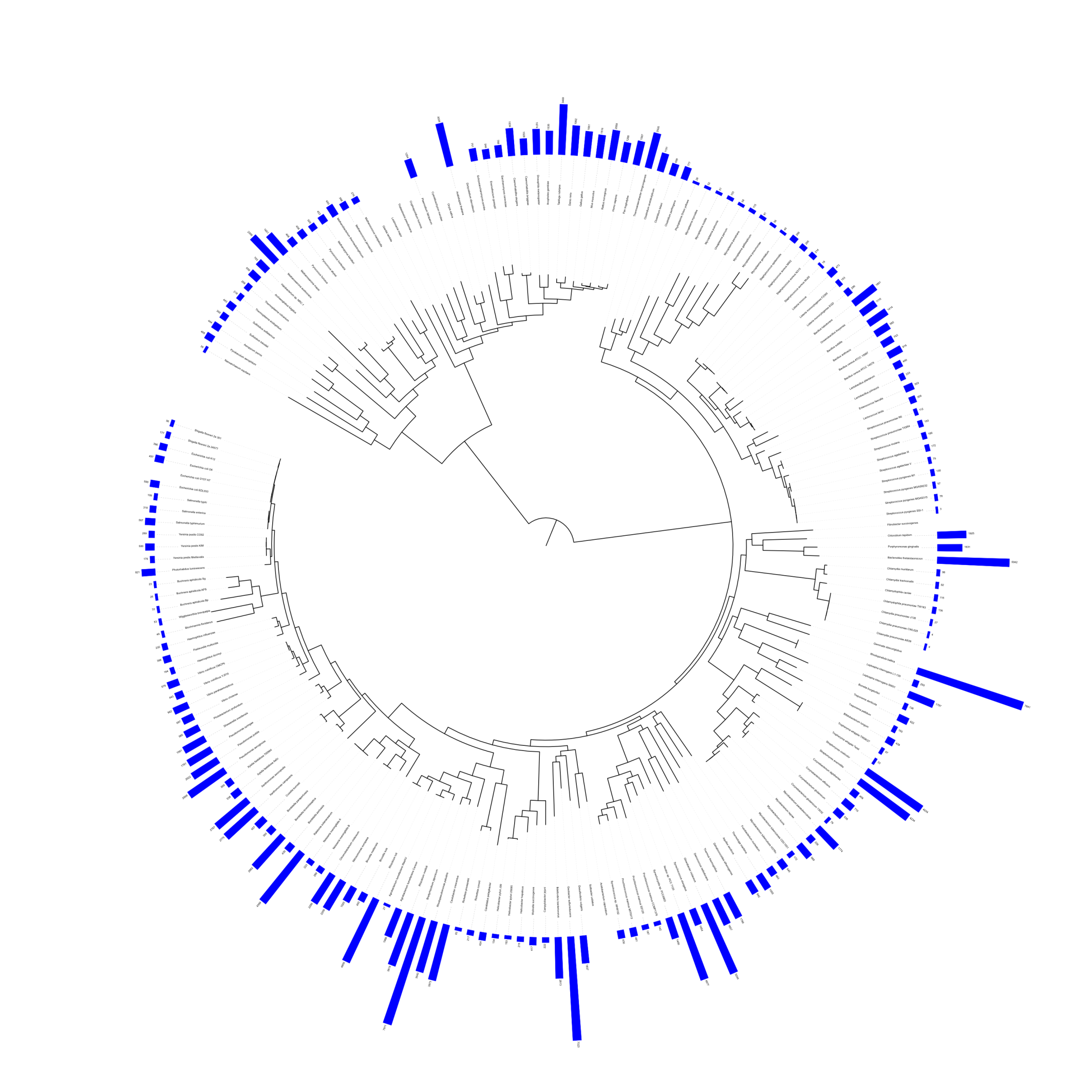

Supplement: Figure S5 — Full phylogenic distribution of the soil set. (18.08 MB TIF) [file pone.0002607.s010.tif]
